# Microwave Assisted Synthesis, Spectral and Antifungal Studies of 2-Phenyl-N,N′-bis(pyridin-4-ylcarbonyl)butanediamide Ligand and Its Metal Complexes

**DOI:** 10.1155/2014/404617

**Published:** 2014-03-19

**Authors:** Rayees Ahmad Shiekh, Maqsood Ahmad Malik, Shaeel Ahmed Al-Thabaiti, Mohmmad Younus Wani, Arshid Nabi

**Affiliations:** ^1^Biomaterial Research Unit, School of Dental Sciences, Universiti Sains Malaysia, Kubang Kerian, 16150 Kelantan, Malaysia; ^2^Department of Chemistry, Faculty of Science, King Abdulaziz University, P.O. Box 80203, Jeddah 21589, Saudi Arabia; ^3^Departamento de Quımica, FCTUC, Universidade de Coimbra, Rua Larga, 3004-535 Coimbra, Portugal; ^4^School of Pharmaceutical Sciences, Lovely Faculty of Applied Medical Sciences, Lovely Professional University, Punjab-144411, India

## Abstract

2-Phenyl-N,N′-bis(pyridin-4-ylcarbonyl)butanediamide ligand with a series of transition metal complexes has been synthesized *via* two routes: microwave irradiation and conventional heating method. Microwave irritation method happened to be the efficient and versatile route for the synthesis of these metal complexes. These complexes were found to have the general composition M(L)Cl_2_/M(L)(CH_3_COO)_2_ (where M = Cu(II), Co(II), Ni(II), and L = ligand). Different physical and spectroscopic techniques were used to investigate the structural features of the synthesized compounds, which supported an octahedral geometry for these complexes. *In vitro* antifungal activity of the ligand and its metal complexes revealed that the metal complexes are highly active compared to the standard drug. Metal complexes showed enhanced activity compared to the ligand, which is an important step towards the designing of antifungal drug candidates.

## 1. Introduction

Nicotinamide is known as a component of the vitamin B complex as well as a component of the coenzyme, nicotinamide adenine dinucleotide (NAD). It is documented that heterocyclic compounds play a significant role in many biological systems, especially N-donor ligand systems being a component of several vitamins and drugs such as nicotinamide [1–3]. The presence of pyridine ring in numerous naturally abundant compounds is also of scientific interest. Nicotinamide itself plays an important role in the metabolism of living cells and some of its metal complexes are biologically active as antibacterial or insulin-mimetic agents [4]. Therefore, the structure of nicotinamide has been the subject of many studies [5–8]. Uses of metal ions in therapeutic agents are known to accelerate drug action and their efficacy enhances upon coordination with a metal ion [9, 10]. The classical coordination complex, cis-DDP or cisplatin (cis-diammine dichloroplatinum), has been the subject of much recent attention towards the metal-based chemotherapy, because of its beneficial effects in the treatment of cancer. These compounds present a great variety of biological activities, namely antitumour [11], antimicrobial [12, 13], anti-inflammatory, and antiviral activities. The inherent biological potential of sulphur/nitrogen donor ligands prompted us to undertake systematic studies with transition metals. In case of N and C based functionalized macrocyclic ligands, the mode of metal incorporation is very much similar to that of metalloproteins in which the requisite metal is bound in a macrocyclic cavity or cleft produced by the conformational arrangement of the protein [14]. The attachment of metal ions to proteins such as monoclonal antibodies can create new tools for use in biology and medicine [15]. These types of ligands have theoretical importance also because they are capable of furnishing an environment with controlled geometry and ligand field strength [16, 17]. The reagents used for such attachments are called bifunctional chelating agents [18]. The precise molecular recognition between macrocyclic ligands and their guest provides a good opportunity for studying key aspects of supramolecular chemistry, which are also significant in a variety of disciplines including chemistry, biology, physics, medicine and related science, and technology [19].

*Candida albicans* is an opportunistic and often deadly pathogen that attacks host tissues, undergoes a dimorphic shift, and then grows as a fungal mass in the kidney, heart, or brain. It is the fourth leading cause of hospital-acquired infection in the United States and over 95% of AIDS patients suffer from infections by* C. albicans* [20, 21].* Candida albicans* is the predominant organism associated with candidiasis; but other* Candida* species, including* C. glabrata*,* C. tropicalis*,* and C. krusei*, are now emerging as serious nosocomial threats to patient populations [22]. The current antifungal therapy suffers from drug related toxicity, severe drug resistance, nonoptimal pharmacokinetics, and serious drug-drug interactions. The common antifungal drugs currently used in clinics belong to polyenes and azoles. Polyenes (amphotericin B and nystatin) cause serious host toxicity [23], whereas azoles are fungistatic and their prolonged use contributes to the development of drug resistance in* C. albicans* and other species [24]. Because of all these striking problems, there is an immediate need to develop novel antifungal drugs with higher efficiency, broader spectrum, improved pharmacodynamic profiles, and lower toxicity. In view of the importance of transition metal complexes in chemotherapy and as part of our continuing interest in metal-complexes, we report herein the synthesis, characterization, and* in vitro* antifungal study of 2-phenyl-N,N′-bis(pyridin-4-ylcarbonyl)butanediamide and its metal complexes.

## 2. Experimental

All the chemicals used were of analytical grade and were procured from Aldrich. Metal salts were purchased from E. Merck and were used as received. All solvents used were of standard/spectroscopic grade. All synthesis and handling were carried out under an atmosphere of dry and oxygen-free nitrogen using standard Schlenk techniques and samples for microanalysis were dried in vacuum to constant weight.

### 2.1. Physical Measurements

Elemental analyses were performed by a Perkin Elmer 2400 CHNSO Elemental Analyser. FT-IR spectra of solid samples were recorded on a Perkin Elmer Spectrum 100 FT-IR spectrometer (Universal/ATR Sampling Accessory). Bruker DPX-300 MHz spectrophotometer was used to record ^1^H NMR spectra at room temperature with DMSO *d*_6_ as solvent. The chemical shift (*δ*) is reported in parts per million (ppm) using tetramethylsilane as internal standard. Positive and negative ESI mass spectra were measured by Bruker (esquire 3000-00037) instrument. Magnetic susceptibility measurements were approved from a microanalysis laboratory by the Gouy method at room temperature. Electronic spectra were recorded on a spectro-UV-Vis Dual Beam 8 auto cell UVS-2700 LABOMED, Inc. US spectrophotometer using DMSO as solvent. Melting points (mp) were recorded on a Metrex melting point apparatus and the results are uncorrected.

### 2.2. Synthesis of Ligand (L)

*2-Phenyl-N,N*′*-bis(pyridin-4-ylcarbonyl)butanediamide. *Two different routes were employed for the synthesis of the ligand.

#### 2.2.1. Microwave Assisted Synthesis

**Scheme 1:**
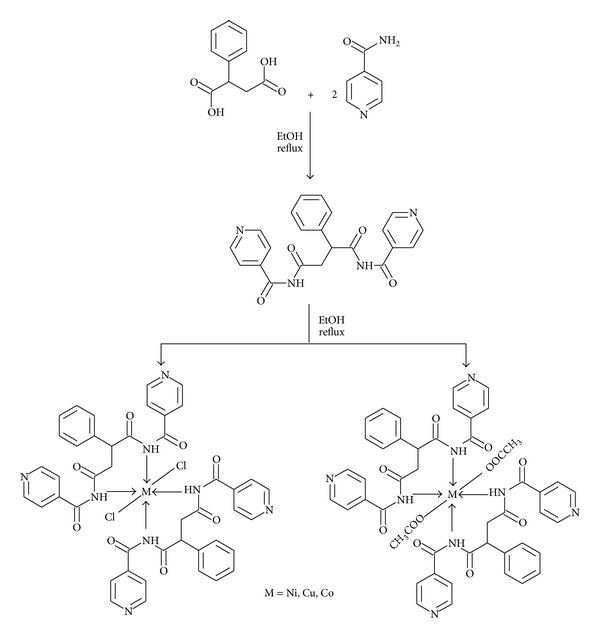
Synthesis of ligand and its metal complexes.

The ligand (L) was prepared by the condensation of phenylsuccinic acid (0.97 g, 5 mmoL) with nicotinamide (1.22 g, 10 mmoL). The reaction mixture was irradiated by the conventional microwave oven by taking 2-3 mL solvent. The reaction was completed in a short period (3-4 min). The resulting precipitate was then recrystallized from alcohol and dried under vacuum. These were characterized and analysed before use. Elemental analyses were conducted using the methods mentioned above and their results were found to be in good agreement with the calculated values. The structure of the ligand has been shown in Scheme 1.

Yield 95%, mp. 180°C, IR (KBr, cm^−1^): 3483 (N–H), 2985 (C–H), 1645 (C=O), 1580 (C–N), 1012, 863, 741; ^1^H NMR (300 MHz, *δ* ppm from TMS in DMSO–*d*_6_, 300 k): *δ* 8.01–7.17 (13H, Ar–C–H), *δ* 11.70–11.62 (2H, OC–NH), *δ* 2.88–2.63 (1H, OC–CH_2_). ESI MS (*m/z*) 402 [M]^+^, 403 [M+1]^+^. Elem anal calcd. C 65.66, H 4.51, N 13.92%; found C 66.22, H 4.75, N 14.01.

#### 2.2.2. Conventional Thermal Method

For comparison purposes, the above ligand was also synthesized by the thermal method. In this method, 100 mL of ethanol was used to dissolve the starting materials of the ligand and the contents were refluxed for nearly 6-7 h. The residue formed was separated out, filtered off, washed with water, recrystallized from ethanol, and finally dried in vacuum over fused calcium chloride (yield 82%), mp. 180°C. A comparison between thermal method and microwave method is given in Table 1.

### 2.3. Synthesis of Metal Complexes 

#### 2.3.1. Microwave Assisted Synthesis

The ligand and metal salts were mixed in 1 : 2 (metal : ligand) ratio in a grinder. The reaction mixture was then irradiated by the microwave oven by taking 3–5 mL solvent. The reaction was completed in a short time (5–7 min) with higher yields. The resulting product was then recrystallized from ethanol and ether and finally dried under reduced pressure over anhydrous CaCl_2_ in a desiccator. The progress of the reaction and purity of product was monitored by TLC using silica gel G.

#### 2.3.2. Conventional Thermal Method

These complexes were also synthesized by the thermal method where instead of 5–7 min, reactions were completed in 4-5 h and the yield of the products was also less than that obtained by the microwave assisted synthesis.

#### 2.3.3. [M(L)Cl_2_/M(L)(CH_3_COO)_2_] Type of Complexes

M(L)Cl_2_/M(L)(CH_3_COO)_2_ (where M = Cu(II), Co(II), Ni(II), and L = ligand).

A hot ethanolic (20 mL) solution of the corresponding metal salts (0.05 mmoL) was added to a hot ethanolic (20 mL) suspension of the macrocyclic ligand (0.10 mmoL). The mixture was stirred for 4-5 hours at 30°C and the solution was reduced to half of its volume. It was then allowed to stand overnight in a refrigerator. A coloured complex precipitated out, which was secluded by filtration under vacuum. It was washed systematically with cold ethanol and dried in vacuum over P_4_O_10_.

### 2.4. Antimicrobial Activity

#### 2.4.1. Yeast Strains, Media, and Growth Conditions

The* Candida* strains were cultured in yeast extract, peptone, and dextrose (YEPD) broth and maintained on YEPD agar plates at 4°C and restreaked every 4–6 weeks. The culture was initiated with a loop full of cells maintained on YEPD slants into a 50 mL of appropriate medium (YEPD) and grown at 37°C in a rotary shaker at 150–170 rpm to the stationary phase (24 h) of growth and for experimental purposes 5 × 10^6^ cells (optical density *A*_595_ = 0.1) were inoculated into the fresh media. Growth was followed for further 24–48 h and measured turbidometrically using LaboMed Spectrophotometer at 595 nm. For long term storage cultures were stored at −20°C with 1 : 1 glycerol as glycerol stocks.

#### 2.4.2. Assessment of the Minimum Inhibitory Concentration (MIC_90_)

Minimum inhibitory concentration (MIC_90_) is defined as the lowest concentration (highest dilution) of the test agent that causes a 90% decrease in absorbance compared with that of control. The MICs of the ligand and its metal complexes against various* Candida* isolates were determined by the broth microdilution method, as described by the Clinical and Laboratory Standard Institute (CLSI) [25] (formerly the National Committee for the Clinical Laboratory Standards) (approved standard M27-A2, 2002). Stock solutions of the test compounds were prepared in DMSO. The cells were grown in YNB medium containing 2% glucose. The diluted cell suspensions were added to the wells of round-bottomed 96-well microtitre plates (100 *μ*L/well) containing equal volumes of medium (100 *μ*L/well) and different concentrations of test compounds. A drug-free control was also included. The plates were incubated at 35°C for 24 h. The MIC test endpoint was also evaluated both visually and by observing OD_595_ in a microplate reader (BIO-RAD, i Mark, US) and is defined as the lowest compound concentration that gave ≥90% inhibition of growth compared with the growth of the controls.

#### 2.4.3. WST-1 Cytotoxicity Assay

*Colorimetric Assay for Quantification of Cellular Cytotoxicity & Proliferation. *This is the accurate method for measuring the cellular toxicity [26]. In this experiment 2.5 mL assay buffer was added directly to each vial of water-soluble tetrazolium salt (WST-1) and cytoscan electron carrier (CEC). The obtained solution was stored at −20°C and protected from light. Equal volumes of the WST-1 and CEC solutions were mixed to prepare the assay dye solution before use and stored at −20°C and protected from light.

For a cytotoxicity assay, 5 × 10^4^–5 × 10^5^ cells were cultured per well of a 96-well plate with a final volume of 100 *μ*L/well culture medium for 24 h. Following incubation, different concentrations of ligand and its metal complexes were exposed to* Candida *cells for a period of 1 h. At the end of the treatment, 10 *μ*L WST-1/ CEC assay dye solution was added to each well and the plate was gently shaken to mix chemicals with medium. The plate was incubated for 30 mins in the incubator. It was then shaken for 1 minute on the shaker and absorbance was measured using a microplate reader at 450 nm and reference was set at wavelength 655 nm. All positive controls (varying concentrations of test compounds) were also included to subtract the reducing activity of test compounds towards tetrazolium from test results. The culture medium background was subtracted from assay results and percentage cytotoxicity was calculated with the following equation, using average absorbances for controls and experimental results as shown earlier [27].

Consider

(1) $$\begin{array}{c} \%\;\text{Cytotoxicity}=\left[\frac{100\times \left(\text{Cell Control}-\text{Experimental}\right)}{\left(\text{Cell Control}\right)}\right]. \end{array}$$

## 3. Results and Discussion

On the basis of elemental analyses, the complexes were assigned the composition shown in Table 2. The analytical data of the complexes correspond well with the general formula [M(L)Cl_2_/M(L)(CH_3_COO)_2_], where L = ligand and M = Co(II), Ni(II), Cu(II). The molar conductance indicates that all the complexes are 1 : 2 electrolytes in nature.

### 3.1. Infrared Spectra

Assignments of selected characteristic IR band positions provide significant indication for the formation of ligand (L) and its complexes. The appearance of new bands in the IR spectra at 1645 *ν*(C=O) amide I, 1580 *ν*(C–N) + *δ*(N–H) amide II, and 1255 *δ*(N–H) amide III are characteristic for amide groups (Table 3). A sharp band observed in the region of 3483 cm^−1^ may be assigned to *ν*(N–H) of the amide group [28].

On complexation, the position of *ν*(N–H) and *υ*(C–N) bands shifting to a lower frequency compared to macrocyclic ligand indicates that the coordination takes place through the nitrogen [N_4_] core and a new medium intensity band appearing at 420–465 cm^−1^ attributed to *ν*(M–N) also designates that the flow of electron density towards the metal atom is through the nitrogen group [29]. Another medium intensity band in the region of 340–360 cm^−1^ has been assigned to *ν*(M–Cl). The presence of bands in the regions of 1595–1560 cm^−1^ and 1330–1315 cm^−1^ are characteristic of asymmetric and symmetric COO^−^ stretching vibrations, respectively, with Δ*ν* = ~250 cm^−1^ [30].

### 3.2. ^1^H NMR Spectra

^1^H NMR spectral data of the ligand in DMSO *d*_6_ shows the signals corresponding to the proposed structure, as it does not show any signal corresponding to the primary amine group and alcoholic proton. The ligand shows a multiplet in the region of 8.01–7.17 ppm Ar–CH (13H), due to the presence of aromatic ring protons. There is a sharp signal in the range of 11.70–11.62 ppm which is attributed to amide CO–NH, (2H) [31–33]. Another signal appearing in the range of 2.88–2.63 ppm has been ascribed to methylene protons OC–CH_2_, (1H). These proton signals undergo down field shifting in all the metal complexes of the ligand because of the paramagnetic effect of metal (II) ions and hence support the coordination of the ligand towards the metal ions [34, 35].

### 3.3. Electrospray Ionization Mass Spectra (ESI MS)

The mass spectra of ligand (L) confirm the proposed formula by showing a peak at* m/z* 403 corresponding to the moiety [(C_22_H_18_N_4_O_4_)^+^] atomic mass* m/z* 402. The series of peaks in the range* m/z* 76, 120.7, 164.6, 230, 349, and so forth may be assigned to various fragments. Their intensity gives an idea of the stability of fragments. [M+2]^+^ peaks were observed in [CoL]Cl_2_, [NiL]Cl_2_, and [CuL]Cl_2_ metal complexes, possibly due to the presence of isotopic chlorine in low quantities [35]. In some cases, the molecular ion peak was also associated with the solvent, water molecules, and some adduct ions from the mobile phase solution [36, 37] (Table 2).

### 3.4. Bands due to Anions

#### 3.4.1. Cobalt(II) Complex

At room temperature the magnetic moment of cobalt(II) complexes lie in the range of 4.82–4.98 B.M. corresponding to three unpaired electrons [38]. The electronic spectra of cobalt(II) complexes exhibit absorption in the region 11,180–11,450 cm^−1^, 14,710–16,680 cm^−1^, 18,550–18,595 cm^−1^, and 25,135–29,750 cm^−1^. These bands may be assigned to ^4^T_1*g*_ (F)→ ^4^T_2*g*_ (F) (*ν*_1_), ^4^T_1*g*_→ ^4^A_2*g*_ (*ν*_2_), and ^4^T_1*g*_ (F)→ ^4^T_1*g*_ (P) (*ν*_3_) transitions, respectively, and the fourth band may be due to charge transfer, suggesting an octahedral geometry around cobalt(II) ion [39].

#### 3.4.2. Copper(II) Complex

The magnetic moment of all the Cu(II) complexes recorded at room temperature lie in the range 1.90–1.99 B.M. corresponding to one unpaired electron. The electronic spectra of the copper(II) complexes display bands in the range 10,263–11,486 cm^−1^, 18,435–18,650 cm^−1^, and 29,433–29,850 cm^−1^. The first two bands may be assigned to the transitions: ^2^B_1*g*_→ ^2^A_1*g*_ (*d*_*x*−*y*2_→*d*_*z*2_) (*ν*_1_) and ^2^B_1*g*_→ ^2^B_2*g*_ (*d*_*x*2−*y*2_→*d*_*zy*_) (*ν*_2_), respectively, and third band is due to charge transfer spectra [40].

#### 3.4.3. Nickel(II) Complex

The magnetic moment of the Ni(II) complex at room temperature lie in the range of 2.75–2.90 B.M. corresponding to two unpaired electrons. The electronic spectra of Ni(II) complexes display three absorption bands in the region of 10,190–10,210 cm^−1^, 18,738–18,757 cm^−1^, and 20,410–21,550 cm^−1^. These bands may be assigned to ^3^A_2*g*_(F)→ ^3^T_2*g*_(F)(*υ*_1_), ^3^A_2*g*_(F)→ ^3^T_1*g*_(F)(*υ*_2_), and ^3^A_2*g*_(F)→ ^3^T_1*g*_(P)(*υ*_3_), respectively [41], showing six coordinated distorted octahedral geometries as shown in Scheme 1.

### 3.5. Antimicrobial Screening

#### 3.5.1. Assessment of Minimum Inhibitory Concentration (MIC_90_)

**Figure 1:**
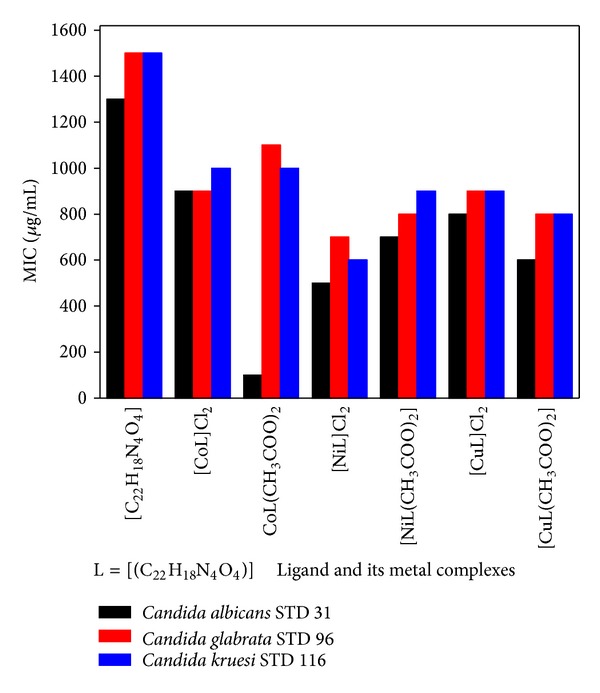
Minimum inhibitory concentration (90%) of the synthesized ligand and its metal complexes against different clinical fluconazole-sensitive* Candida *isolates.

Figure 1 summarizes the* in vitro* susceptibilities of 3 fluconazole-sensitive* Candida* strains against ligand and its metal complexes. The data is reported as MIC which is defined as the lowest concentration required inhibiting 90% growth in comparison to control (absence of any test compound) for each isolate. The synthesized compounds were found to be active against all the tested* Candida* isolates. The MIC_90_ of ligand [C_22_H_18_N_4_O_4_] against the sensitive isolates of different* Candida* isolates ranged 1300–1500 *μ*g/mL, [CoL]Cl_2_ ranged 900–1000 *μ*g/mL, [CoL(CH_3_COO)_2_] ranged 1000–1100 *μ*g/mL, [NiL]Cl_2_ ranged 500–700 *μ*g/mL, [NiL(CH_3_COO)_2_]Cl_2_ ranged 700–900 *μ*g/mL, [CuL]Cl_2_ ranged 800–900 *μ*g/mL, and that of [CuL(CH_3_COO)_2_] ranged 600–800 *μ*g/mL, respectively.

#### 3.5.2. Growth Curve Studies

**Figure 2:**
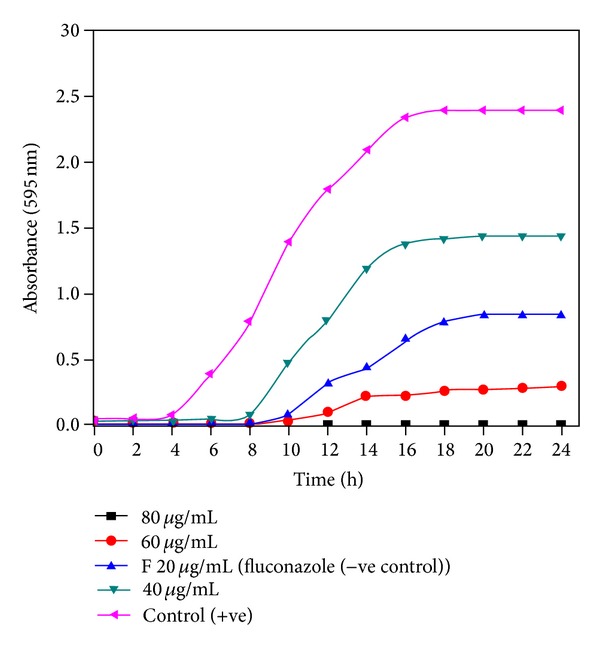
Effect of NiCl_2_ complex in the concentration range of 40–80 *μ*g/mL was studied on* C. albicans* STD 31. Growth curve plotted against absorbance at 595 nm and time (h) shows complete inhibition of growth at 80 *μ*g/mL.

In the case of growth curve studies, the effect of increasing concentrations of the ligand and its complexes on the growth pattern of different fungal species has been studied. Control cells showed a normal pattern of growth with a lag phase of 4 h and an active exponential phase of 8–10 h before attaining stationary phase. An increase in the concentration of test compounds leads to a significant decrease in growth. NiCl_2_ complex when treated against* Candida albicans* at a concentration of 40 *μ*g/mL the growth pattern does not change, the lag phase is extended by 4 h, and the stationary phase does not reach the same level of cell growth as in the case of control, and at 60 *μ*g/mL the lag phase is further extended by 2 h. At a concentration of 80 *μ*g/mL (MIC_90_ level), there is a total inhibition of growth showing a flat line (Figure 2). Fluconazole 20 *μ*g/mL showed the lag phase further extended by 6 h with respect to control. A significant and pronounced effect is observed for all the synthesized complexes. Ni(II), Cu(II), and Co(II) complexes, in a concentration dependent manner, suppressed growth and delayed exponential phases. At MIC_90_ values complete inhibition of growth was observed.

#### 3.5.3. WST-1 Cytotoxicity Assay

The assay principle is based upon the reduction of the tetrazolium salt (WST-1) to formazan by cellular dehydrogenases that can be assessed visually and quantified spectrophotometrically. The generation of yellow coloured formazan is measured at 450 nm and is directly correlated to cell number.  Table 4 gives % cytotoxicity of* C. albicans* STD 31,* Candida glabrata* STD 96, and* Candida kruesi* STD 116 at MIC concentrations of test compounds. All the test compounds show cytotoxicity for all the three fluconazole-susceptible* Candida* isolates used in this study. It was found that MIC_90_ values of the test compounds showed pronounced cytotoxic effects. The average % cytotoxicity at MIC values of [CoL]Cl_2_, [CoL(CH_3_COO)_2_], [NiL]Cl_2_, [NiL(CH_3_COO)_2_]Cl_2_, [CuL]Cl_2_, and [CuL(CH_3_COO)_2_] was 44%, 35%, 86%, 69%, 59%, and 76% against the three types of species. Respective figures at the MIC values of ligand [C_22_H_18_N_4_O_4_] were 20% only. [NiL]Cl_2_ at its MIC concentrations was found to be the most cytotoxic of all the compounds tested. An increase in cytotoxicity was observed with increase in concentration of the test compounds.

## 4. Conclusion

The synthesis and characterization of 2-phenyl-N,N′-bis(pyridin-4-ylcarbonyl)butanediamide ligand and its corresponding Cu(II), Co(II), and Ni(II) complexes have been carried out. The IR, ^1^H-NMR, and ^13^C-NMR data were successfully used to elucidate the formation of the 2-phenyl-N,N′-bis(pyridin-4-ylcarbonyl)butanediamide ligand. On the basis of spectral studies an octahedral geometry for metal complexes has been assigned. All the fluconazole-susceptible* Candida* isolates investigated were found to be sensitive to the test compounds. The use of total mean MICs obtained gave a good indication of the overall antimicrobial effectiveness of each test compound. This may indicate that the yeast physiology may not be better equipped to counteract the antifungal properties of these compounds. The higher activity of [NiL]Cl_2_ as compared to free ligand [C_22_H_18_N_4_O_4_] may be attributed to the increased lipophillicity that causes its efficient permeation through the lipid bilayers of the microbial cell membranes and a consequent cell death [42]. Cytotoxicity results obtained suggest that there is a drastic alteration in redox activity of cells and at higher values of metal chelates a maximum decrease in reduction of tetrazolium salt is seen. By combining the results of MIC studies and tetrazolium assays it can be concluded that metal chelates at their MIC_90_ values show maximum effect either on growth or on metabolic activities of oxidases inside the cell. At higher concentrations, these metal chelates affected redox activity translates decreased growth which eventually leads to maximum growth inhibition at MIC_90_ values.

## Figures and Tables

**Table 1:** Comparison between microwave and thermal method.

Compound	Yield (%)		Solvent (mL)		Time	
	Thermal	Microwave	Thermal	Microwave	Thermal (h)	Microwave (min)
[C_22_H_18_N_4_O_4_]	82	95	100	2	7	3
[CoLCl_2_]	75	87	40	4	5	5
[CoL(CH_3_COO)_2_]	76	86	40	3	5	6
[NiLCl_2_]	78	85	40	5	5	5
[NiL(CH_3_COO)_2_]	72	82	40	4	4	6
[CuLCl_2_]	69	79	40	4	5	7
[CuL(CH_3_COO)_2_]	73	83	40	3	4	6

L = [(C_22_H_18_N_4_O_4_)].

**Table 2:** Analytical data and physical properties of the ligand and complexes.

Complexes molecular formula	Colour	Molar conductance (Ω^−1^cm^2^ mol^−1^)	M.P. (°C)	Mol. Wt. found (Cal.) %	Molecular weight found (calculated) %				
					M	C	H	N	O
[C_22_H_18_N_4_O_4_]	Colourless		180	402 (401.45)		65.66 (66.02)	4.51 (4.75)	13.92 (14.01)	15.90 (15.98)
[CoL]Cl_2 _	Pink	263	220	531.83 (503.78)	11.08 (11.05)	49.63 (49.58)	3.38 (3.35)	10.52 (10.49)	12.03 (12.01)
[CoL(CH_3_COO)_2_]	Mauve pink	205	210	578.93 (577.95)	10.17 (10.14)	45.60 (45.55)	3.10 (3.08)	9.67 (9.64)	11.05 (11.02)
[NiL]Cl_2_	Green	220	215	531.59 (530.69)	11.04 (11.01)	49.66 (49.62)	3.38 (3.36)	10.53 (10.50)	12.03 (12.00)
[NiL(CH_3_COO)_2_]	Light green	202	208	578.69 (577.89)	10.14 (10.10)	45.62 (45.58)	3.11 (3.09)	9.67 (9.65)	11.05 (11.02)
[CuL]Cl_2 _	Royal blue	258	218	536.44 (535.24)	11.84 (11.80)	49.21 (49.18)	3.35 (3.31)	10.43 (10.40)	11.93 (11.90)
[CuL(CH_3_COO)_2_]	Greenish blue	198	212	583.54 (582.24)	10.88 (10.82)	45.24 (45.20)	3.08 (3.05)	9.59 (9.56)	10.96 (10.93)

L = [(C_22_H_18_N_4_O_4_)].

**Table 3:** Relevant IR spectral peaks (cm^−1^) and their assignments.

Complexes	*ν* N–H	Amide-I [*ν* C=O]	Amide-II [*ν* C–N, *δ* N–H]	Amide-III [*δ* N–H]	*ν* M–N	*ν* M–Cl	*ν* M–(CH_3_COO)	
							(asym)	(sym)
[C_22_H_18_N_4_O_4_]	3483	1645	1580	1255				
[CoL]Cl_2 _	3250	1632	1565	1232	445	340	—	
[CoL(CH_3_COO)_2_]	3165	1640	1552	1225	456	—	1564	1325
[NiL]Cl_2_	3153	1638	1558	1242	459	354	—	
[NiL(CH_3_COO)_2_]	3147	1625	1569	1220	454	—	1595	1315
[CuL]Cl_2 _	3150	1630	1570	1238	425	350	—	
[CuL(CH_3_COO)_2_]	3145	1628	1563	1222	432	—	1560	1330

L = [(C_22_H_18_N_4_O_4_)].

**Table 4:** % Cytotoxicity by MIC of ligand and its different complexes against three fluconazole-sensitive *Candida* isolates.

Complexes (µg/mL)	Control	*Candida albicans *STD 31	*Candida glabrata* STD 96	*Candida kruesi* STD 116
		0	0	0
[C_22_H_18_N_4_O_4_]	MIC	24.7 ± 0.2	20.2 ± 0.8	18.4 ± 1.1
[CoL]Cl_2 _	MIC	48.5 ± 0.1	45.2 ± 0.2	41.5 ± 0.7
[CoL(CH_3_COO)_2_]	MIC	41.1 ± 0.4	35.0 ± 0.9	30.7 ± 0.3
[NiL]Cl_2_	MIC	89.6 ± 0.7	82.1 ± 0.4	88.2 ± 0.6
[NiL(CH_3_COO)_2_]	MIC	71.0 ± 0.9	70.8 ± 0.8	67.1 ± 0.9
[CuL]Cl_2 _	MIC	63.0 ± 0.2	59.5 ± 0.5	57.2 ± 0.7
[CuL(CH_3_COO)_2_]	MIC	78.2 ± 0.6	74.1 ± 0.7	77.0 ± 0.3

L = [(C_22_H_18_N_4_O_4_)].
